# Cerebral Hemodynamic Changes and Postoperative Brain Function in Patients Undergoing Cardiac Valve Surgery With Cardiopulmonary Bypass: A Prospective Cohort Study

**DOI:** 10.7759/cureus.91327

**Published:** 2025-08-31

**Authors:** Changxue Wu, Zhi Wen, Zheng Wang, Chen Chen, Yangyun Han, Xiaoxiao Xie, Jianqiang Wu, Mingwu Tian

**Affiliations:** 1 Department of Cardiothoracic Vascular Surgery, Deyang People's Hospital, Deyang, CHN; 2 Department of Neurosurgery, Deyang People's Hospital, Deyang, CHN; 3 Department of Neurology, Deyang People's Hospital, Deyang, CHN

**Keywords:** cardiac valve surgery, cardiopulmonary bypass, cerebral hemodynamics, postoperative delirium, transcranial doppler

## Abstract

Background

Cardiopulmonary bypass (CPB) during cardiac surgery disrupts cerebral hemodynamics, potentially leading to postoperative neurological complications such as delirium. Transcranial Doppler (TCD) offers non-invasive monitoring of cerebral blood flow alterations, yet its predictive value for delirium remains underexplored. This study aims to investigate the association between intraoperative cerebral hemodynamic changes monitored by TCD and postoperative delirium in patients undergoing cardiac valve surgery with CPB.

Methods

This prospective cohort study enrolled 184 elective cardiac valve surgery patients. TCD measured middle cerebral artery mean flow velocity (Vm), pulsatility index (PI), and resistance index (RI) at five perioperative time points: 24 hours preoperative (T1), five minutes post anesthesia induction (T2), five minutes intraoperative post-cross-clamping (T3), five minutes post resumption of cardiac beating (T4), and prior to operating room discharge (T5). Delirium was diagnosed using the Confusion Assessment Method for the ICU (CAM-ICU). Neuropsychological assessments using Richmond Agitation-Sedation Scale (RASS) and Mini-Mental State Examination (MMSE) were conducted preoperatively and at 24 and 48 hours postoperatively.

Results

Postoperative delirium occurred in 20 patients (10.9%). The delirium group demonstrated significantly longer CPB duration (143.2 ± 19.7 minutes versus 113.8 ± 16.4 minutes; P < 0.001) and aortic cross-clamp time (97.6 ± 15.3 minutes versus 72.4 ± 12.1 minutes; P < 0.001). Both groups exhibited cerebral hypoperfusion during CPB (T3-T4 versus T1: reduced Vm, elevated PI and RI; P < 0.05), with more severe disturbances in delirium patients (T3 Vm: 25.8 ± 3.7 cm/s versus 29.5 ± 4.0 cm/s; P < 0.001). Prior to operating room discharge (T5), delirium patients showed persistent hemodynamic abnormalities (Vm: 56.2 ± 5.0 cm/s versus 62.8 ± 5.2 cm/s; PI: 0.96 ± 0.07 versus 0.85 ± 0.06; P < 0.001) while non-delirium patients returned to baseline. Significant cognitive decline was observed in delirium patients (24-hour MMSE: 21.8 ± 2.1 versus 27.2 ± 1.4; P < 0.001).

Conclusion

Intraoperative TCD-detected cerebral hypoperfusion, characterized by reduction in Vm and elevated PI and RI, and prolonged CPB duration are independent predictors of postoperative delirium. Failure of postoperative cerebral hemodynamic recovery may contribute to the pathogenesis of delirium.

## Introduction

Cardiopulmonary bypass (CPB) is a pivotal technique in cardiac surgery that temporarily assumes the functions of the heart and lungs to create a bloodless surgical field, significantly enhancing the feasibility and safety of complex procedures. However, CPB also profoundly disrupts cerebral hemodynamics due to non-pulsatile flow, hypothermia, hemodilution, and microemboli formation [1], predisposing patients to postoperative neurological complications such as delirium and cognitive dysfunction [2,3]. Delirium, an acute, reversible cognitive impairment common in elderly patients after cardiac surgery [4], arises from multifactorial mechanisms, including cerebral hypoperfusion, microembolic events, inflammatory responses, and metabolic disturbances [5]. Crucially, abnormal cerebral hemodynamics, marked by reduced cerebral blood flow (CBF) and increased cerebrovascular resistance (CVR), represent key pathophysiological foundations of delirium [6]. Given the central role of these hemodynamic alterations, real-time assessment through transcranial Doppler (TCD) ultrasound may help elucidate the underlying pathogenesis. TCD offers a non-invasive, real-time method for assessing cerebral hemodynamics by measuring flow velocity in arteries, such as the middle cerebral artery (MCA), thereby indirectly reflecting CBF and CVR [7]. Although TCD is routinely used in cardiac surgery to monitor cerebral perfusion during and after CPB and to detect ischemia or hyperperfusion [8,9], its predictive value for delirium remains underexplored, with existing studies limited by a lack of prospective designs, insufficient multi-time-point assessments, and inadequate correlation with clinical outcomes.

Given the established impact of CPB on cerebral hemodynamics and the close relationship between delirium and abnormal hemodynamic changes, this study utilized TCD to monitor cerebral hemodynamic parameters, including mean flow velocity (Vm), pulsatility index (PI), and resistance index (RI), at multiple perioperative time points in patients undergoing cardiac valve surgery. The primary objective was to investigate the association between these parameters and the occurrence of postoperative delirium. Through multi-time-point monitoring (preoperative, intraoperative, and postoperative), this study aimed to elucidate the potential role of cerebral hemodynamic alterations in the pathogenesis of delirium, while acknowledging that these changes may reflect broader vulnerability rather than solely direct causal mechanisms, particularly in the context of other unmeasured perioperative factors.

## Materials and methods

Patients

This prospective cohort study included 184 consecutive patients who underwent elective cardiac valve surgery with CPB at our institution between March 2022 and November 2024. The study was approved by the Ethics Committee of Deyang People's Hospital (Approval Number: 2022-04-018-K01), and written informed consent was obtained from all participants.

The inclusion criteria were as follows: (1) age ≥ 18 years; (2) scheduled to undergo elective, first-time cardiac valve replacement or repair surgery requiring CPB; (3) the patient or their legal guardian understands the study purpose, procedures, and potential risks, and voluntarily provides written informed consent; (4) preoperative consciousness is clear, allowing cooperation with assessments; (5) adequate transtemporal ultrasound signal quality, enabling clear detection of bilateral MCA blood flow signals.

The exclusion criteria were as follows: (1) Preoperative impairment of consciousness or cognition; (2) history of severe neurological disorders such as stroke, traumatic brain injury, neurodegenerative diseases, intracranial space-occupying lesions, symptomatic severe carotid, or intracranial artery stenosis/occlusion; (3) severe hearing, visual, or speech impairments, or significant language comprehension difficulties affecting assessments; (4) poor temporal window penetration on TCD monitoring, or open wounds, infections, or severe deformities at the probe placement site; (5) active severe psychiatric disorders, chronic alcoholism, or history of drug abuse/dependence; (6) emergency or salvage surgery; (7) anticipated inability to complete postoperative follow-up assessments; (8) concurrent end-stage renal disease or severe hepatic failure; (9) preoperative long-term use of high-dose sedative-hypnotics, antipsychotics, or anticholinergic drugs.

Management of anesthesia and cardiopulmonary bypass

Following overnight fasting, all medications except antihypertensives and levothyroxine were withheld. Anesthesia induction utilized sufentanil 0.5-1 μg/kg, etomidate 0.2 mg/kg, and midazolam 2 mg. Rocuronium provided neuromuscular blockade with 0.8-1 mg/kg at induction and 30 mg during sternal closure. Anesthesia maintenance was achieved with propofol, ciprofol, or remimazolam. Antibiotic prophylaxis was administered 30 minutes prior to skin incision. Before initiating CPB, heparin 300-400 IU/kg was administered, targeting an activated clotting time of ≥480 seconds. A membrane oxygenator delivered nonpulsatile flow at 60-80 ml/(kg·min), with oxygen flow adjusted to 50-67% of perfusion volume using pH-stat blood gas management. Physiological targets included mean arterial pressure (MAP) of 50-80 mmHg, hemodilution to hematocrit of 0.25-0.27, and mild hypothermia maintaining nasopharyngeal temperature at 32-34°C. Myocardial protection employed either 4:1 blood cardioplegia at 20 ml/kg initially, followed by 10 ml/kg every 30 minutes via aortic root or coronary ostia, or histidine-tryptophan-ketoglutarate (HTK) crystalloid solution with an initial 2000 mL dose via direct coronary perfusion, supplemented by blood cardioplegia after two hours if needed. Post bypass, protamine sulfate neutralized residual heparin until the activated clotting time returned to the pre-bypass baseline.

Cerebral blood flow monitoring

TCD (Delica Medical Equipment Co., Ltd., Shenzhen, China) examinations were performed using a 2-MHz probe at five designated time points: 24 hours preoperative (T1), five minutes post anesthesia induction (T2), five minutes intraoperative post-cross-clamping (T3), five minutes post resumption of cardiac beating (T4), and prior to operating room discharge (T5). The optimal signal side, either the right or left cerebral hemisphere, was selected for monitoring. Patients were positioned supine with their heads maintained in the neutral position or adjusted slightly as needed. The probe was placed over the squamous part of the temporal bone within the region 1-5 cm anterior to the ear and superior to the zygomatic arch. The probe was held perpendicular to the cranial surface. Probe position and angle were adjusted to obtain an optimal flow signal from the MCA. The monitoring depth was typically set between 40 mm and 60 mm. The color flow scale and color gain were adjusted to achieve an appropriate signal-to-noise ratio to ensure clear visualization of the flow signal. The following parameters of the MCA were recorded: Vm, PI, and RI. All examinations were performed by physicians with extensive experience in cerebrovascular ultrasound to ensure the accuracy and reproducibility of the measurements.

Postoperative brain function assessment

Postoperative cerebral function was assessed using the Confusion Assessment Method for the Intensive Care Unit (CAM-ICU) [10] to detect delirium, and based on the occurrence of postoperative delirium, patients were divided into two groups: group A (20 patients with delirium) and group B (164 patients without delirium). Cognitive function was evaluated using the Mini-Mental State Examination (MMSE) [11], while sedation levels and agitation were assessed using the Richmond Agitation-Sedation Scale (RASS) [12]. All assessments were conducted preoperatively as well as 24 hours and 48 hours postoperatively. All three scales employed in this study, i.e., the MMSE, RASS, and CAM-ICU, are openly accessible without licensing restrictions.

Data collection and analysis

Demographic and clinical data were collected, including age, sex, body mass index (BMI), comorbidities, types of valve surgery, MAP during CPB, CPB time, and aortic cross-clamp time. CBF data, including Vm, PI, and RI, were collected, and postoperative neurological function was assessed using RASS and MMSE scores. Statistical analysis was performed using SPSS version 25.0 (IBM Corp., Armonk, NY). Continuous variables were expressed as mean ± standard deviation (SD) and compared using repeated-measures analysis of variance (ANOVA) or independent samples t-tests. Categorical variables were expressed as frequencies and percentages, and compared using chi-square tests; if the expected frequency was less than five, Fisher's exact test was used. A p-value < 0.05 was considered statistically significant.

## Results

Baseline characteristics

Among 184 patients undergoing valve surgery, 20 (10.9%) developed postoperative delirium (group A) and 164 (89.1%) did not (group B), with both cohorts demonstrating closely matched baseline profiles (Table 1). Demographic parameters, including age (61.5 ± 4.3 vs. 61.0 ± 5.1 years) and sex distribution (60.0% vs. 61.0% male), comorbid conditions such as atrial fibrillation (20.0% vs. 21.3%), left ventricular dysfunction (15.0% vs. 16.5%), diabetes mellitus (15.0% vs. 15.2%), and hypertension (30.0% vs. 27.4%), along with equivalent valve procedure distributions (aortic: 45.0% vs. 45.1%; mitral: 35.0% vs. 36.0%; combined: 20.0% vs. 18.9%) showed no statistically significant divergence (all P > 0.68). However, patients with delirium exhibited significantly prolonged cardiopulmonary bypass times (143.2 ± 19.7 minutes vs. 113.8 ± 16.4 minutes; P < 0.001) and aortic cross-clamp durations (97.6 ± 15.3 minutes vs. 72.4 ± 12.1 minutes; P < 0.001).

**Table 1:** Comparison of baseline characteristics between delirium and non-delirium groups (mean ± SD, n (%)). ^※^ Fisher's exact test was employed when expected frequencies were less than five, with no χ² values reported. BMI: body mass index; LV: left ventricle; MAP: mean arterial pressure; CPB: cardiopulmonary bypass; ACC: aortic cross-clamp.

Variable	Group A (with delirium, n = 20)	Group B (without delirium, n = 164)	Statistic	P-value
Age (years)	61.5 ± 4.3	61.0 ± 5.1	t = 0.41	0.685
BMI (kg/m²)	25.6 ± 2.6	25.3 ± 2.9	t = 0.37	0.712
Male sex	12 (60.0%)	100 (61.0%)	χ² = 0.01	0.932
Comorbidities				
Atrial fibrillation	4 (20.0%)	35 (21.3%)	Fisher※	0.889
LV dysfunction	3 (15.0%)	27 (16.5%)	Fisher※	0.857
Diabetes mellitus	3 (15.0%)	25 (15.2%)	Fisher※	0.923
Hypertension	6 (30.0%)	45 (27.4%)	χ² = 0.06	0.802
Valve procedure type				
Mitral valve	9 (45.0%)	74 (45.1%)	Fisher※	0.982
Aortic valve	7 (35.0%)	59 (36.0%)		
Combined valves	4 (20.0%)	31 (18.9%)		
MAP during CPB (mmHg)	68.4 ± 7.2	67.8 ± 6.5	0.378	0.706
CPB time (min)	143.2 ± 19.7	113.8 ± 16.4	t = 6.41	<0.001
ACC time (min)	97.6 ± 15.3	72.4 ± 12.1	t = 7.10	<0.001

Cerebral blood flow changes

**Figure 1:**
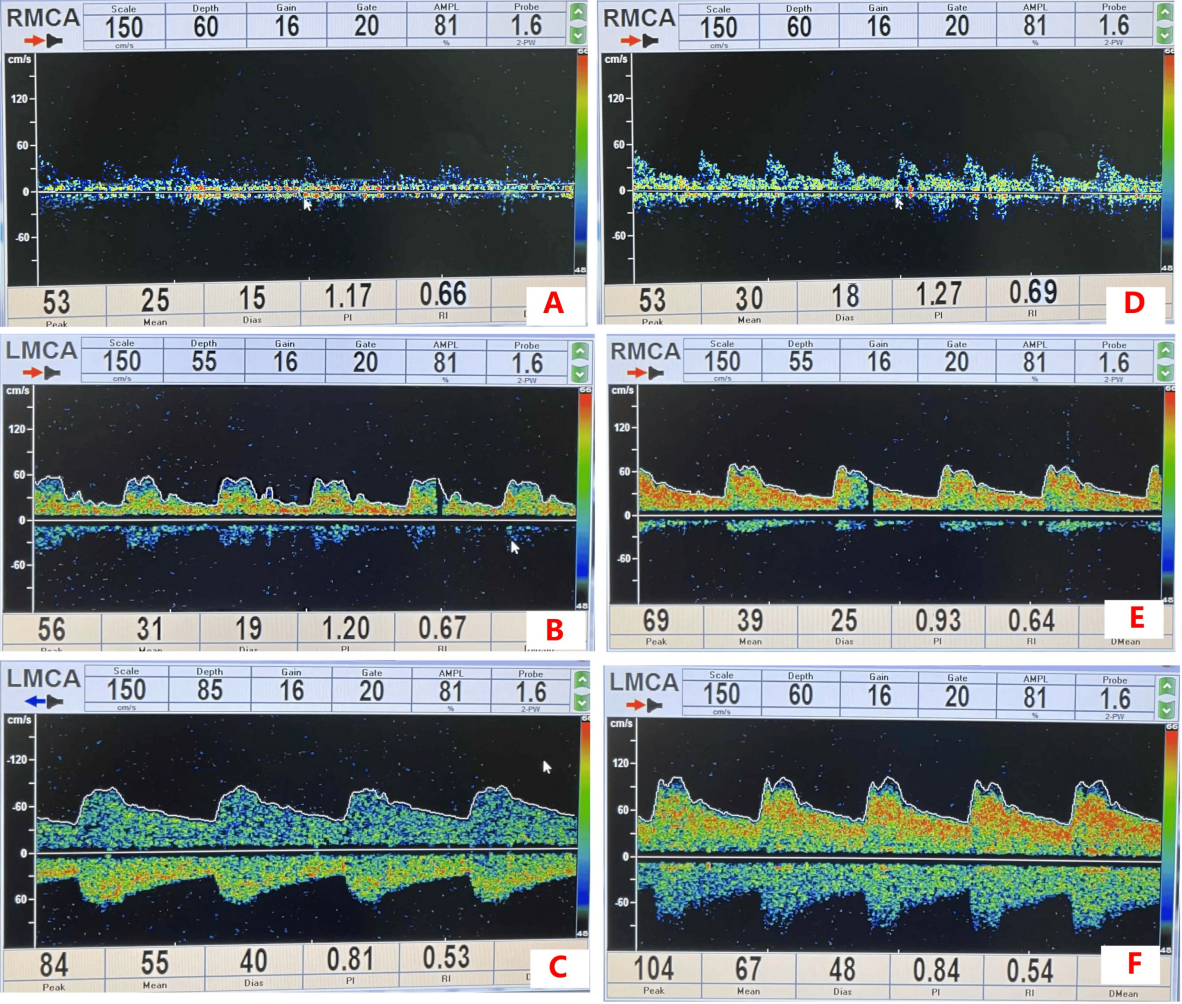
Samples of transcranial Doppler (TCD) flow images in the middle cerebral artery. (A-C) Flow images of group A at T3, T4, and T5. (D-F) Flow images of group B at T3, T4, and T5. LMCA: left middle cerebral artery; RMCA: right middle cerebral artery.

TCD monitoring (Table 2 and Figure 1) demonstrated that at preoperative baseline (T1) and five minutes post-induction (T2), no differences existed in Vm, PI, or RI (all P > 0.05). Following aortic cross-clamping (T3), group A demonstrated lower Vm (25.8 ± 3.7 cm/s vs. 29.5 ± 4.0 cm/s; P < 0.001), higher PI (1.25 ± 0.11 vs. 1.02 ± 0.08; P < 0.001), and elevated RI (0.72 ± 0.05 vs. 0.63 ± 0.04; P < 0.001) compared to group B. These disparities persisted at five minutes after resumption of cardiac beating (T4), with group A showing reduced Vm (32.8 ± 3.8 cm/s vs. 37.5 ± 4.3 cm/s; P < 0.001), increased PI (1.12 ± 0.09 vs. 0.94 ± 0.07; P < 0.001), and higher RI (0.66 ± 0.04 vs. 0.58 ± 0.03; P < 0.001). Prior to operating room discharge (T5), group A maintained lower Vm (56.2 ± 5.0 cm/s vs. 62.8 ± 5.2 cm/s; P < 0.001), elevated PI (0.96 ± 0.07 vs. 0.85 ± 0.06; P < 0.001), and higher RI (0.59 ± 0.04 vs. 0.54 ± 0.03; P < 0.001). Temporally, all parameters at T3 and T4 significantly deviated from preoperative baselines in both groups (P < 0.05), while at T5, only group A exhibited persistent differences from baseline in all hemodynamic parameters (P < 0.05).

**Table 2:** Comparison of TCD parameters between groups at different time points (mean ± SD, n (%)). ^▲^ The comparison of hemodynamic parameters at T3 and T4 versus T1 showed statistically significant differences in both groups (P < 0.05). ^#^ Group A showed statistically significant differences in hemodynamic parameters between T5 and T1 (P < 0.05), while no statistically significant differences were observed in group B (P > 0.05). TCD: transcranial Doppler; ACC: aortic cross-clamping, RCB: resumption of cardiac beating, OR: operating room; Vm: mean flow velocity; PI: pulsatility index; RI: resistance index.

Parameter	Group	T1 (24-hour pre-op)	T2 (5-min post-induction)	T3 (5-min post-ACC)	T4 (5-min post-RCB)	T5 (prior to OR discharge)
Vm (cm/s)	Group A (n = 20)	62.3 ± 5.1	61.8 ± 4.9	25.8 ± 3.7▲	32.8 ± 3.8▲	56.2 ± 5.0#
	Group B (n = 164)	63.1 ± 4.8	62.4 ± 5.0	29.5 ± 4.0▲	37.5 ± 4.3▲	62.8 ± 5.2
	t-value	-0.67	-0.52	-3.78	-4.15	-5.28
	P-value	0.504	0.603	<0.001	<0.001	<0.001
PI	Group A (n = 20)	0.84 ± 0.05	0.85 ± 0.06	1.25 ± 0.11▲	1.12 ± 0.09▲	0.96 ± 0.07#
	Group B (n = 164)	0.83 ± 0.06	0.84 ± 0.05	1.02 ± 0.08▲	0.94 ± 0.07▲	0.85 ± 0.06
	t-value	0.82	0.75	9.06	8.43	8.15
	P-value	0.414	0.455	<0.001	<0.001	<0.001
RI	Group A (n = 20)	0.54 ± 0.04	0.55 ± 0.03	0.72 ± 0.05▲	0.66 ± 0.04▲	0.59 ± 0.04#
	Group B (n = 164)	0.53 ± 0.03	0.54 ± 0.04	0.63 ± 0.04▲	0.58 ± 0.03▲	0.54 ± 0.03
	t-value	0.78	0.91	8.92	8.90	5.48
	P-value	0.436	0.364	<0.001	<0.001	<0.001

Postoperative brain function

A total of 20 patients (10.87%) developed postoperative delirium, classified as group A. Neuropsychological assessment results (Table 3) revealed that preoperative sedation scores (RASS) and cognitive function (MMSE) showed no intergroup differences (both P > 0.4). By 24 hours postoperatively, delirium patients developed hyperactive agitation (RASS: +1.7 ± 0.6 vs. -0.3 ± 0.4; P < 0.001) and significant cognitive impairment (MMSE: 21.8 ± 2.1 vs. 27.2 ± 1.4; P < 0.001). These deficits persisted at 48 hours despite partial recovery (MMSE: 24.3 ± 1.9 vs. 27.8 ± 1.3; P < 0.001), while group B maintained stable neurological function at all time points (P > 0.05).

**Table 3:** Comparison of neuropsychological assessments between groups (mean ± SD). RASS scale: -5 (coma) to +4 (combative), 0 = alert and calm. MMSE scale: 0-30 (higher scores indicate better cognitive function), intact cognition: ≥27 points, mild cognitive impairment: 21-26 points, moderate cognitive impairment: 10-20 points, severe cognitive impairment: ≤9 points. RASS: Richmond Agitation-Sedation Scale; MMSE: Mini-Mental State Examination.

Assessment tool	Group	Preoperative	24-hour postoperative	48-hour postoperative
RASS	Group A (n = 20)	-0.1 ± 0.4	+1.7 ± 0.6	+1.0 ± 0.5
	Group B (n = 164)	-0.2 ± 0.3	-0.3 ± 0.4	-0.1 ± 0.3
	t-value	1.09	18.50	11.20
	P-value	0.278	<0.001	<0.001
MMSE	Group A (n = 20)	28.6 ± 1.1	21.8 ± 2.1	24.3 ± 1.9
	Group B (n = 164)	28.4 ± 1.2	27.2 ± 1.4	27.8 ± 1.3
	t-value	0.76	-12.60	-9.80
	P-value	0.448	<0.001	<0.001

## Discussion

Our TCD monitoring revealed phase-specific cerebral hemodynamic fluctuations during CPB. No significant changes in CBF parameters (Vm, PI, and RI) occurred post anesthesia induction (T2). However, significant reductions in Vm concurrent with elevations in PI and RI were observed during aortic cross-clamping (T3) and after resumption of cardiac beating (T4). This pattern aligns with CPB pathophysiology, wherein non-pulsatile flow, hypothermia, and impaired cerebral autoregulation may increase CVR, while the abrupt decline in cerebral perfusion pressure following aortic clamping exacerbates cerebral hypoperfusion. Furthermore, CPB-generated gaseous microemboli can deform and bypass arterial filters [13], leading to capillary occlusion and focal cerebral ischemia [14]. These microemboli also promote endothelial dysfunction, vascular inflammation, and coagulopathy, which may further alter CVR through endothelial injury and vasoconstrictor release [15].

By the end of surgery (T5), non-delirium patients (group B) regained baseline hemodynamics, whereas delirium patients (group A) exhibited persistently reduced Vm and elevated PI, suggesting impaired cerebral perfusion recovery that may contribute to delirium pathogenesis. Group A showed significantly greater intraoperative Vm reductions (e.g., T3: 25.8 ± 3.7 vs. 29.5 ± 4.0 cm/s) and higher PI/RI values than group B. A >15% decrease in Vm was associated with a 4.2-fold increased risk of delirium, consistent with previous studies linking similar flow reductions to cognitive decline [16]. Moreover, group A’s Vm remained 10.4% below baseline post surgery, implying that delayed reperfusion may underlie delirium pathophysiology.

Delirium was assessed using the CAM-ICU [17], which has high diagnostic validity (sensitivity = 0.73-0.94, specificity = 0.81-0.93) [18]. Group A showed significantly higher RASS scores (+1.7 ± 0.6 vs. -0.3 ± 0.4, p < 0.001) and a 19.9% decline in MMSE scores at 24 hours, exceeding clinically significant thresholds [19]. The MMSE requires interpretation within a clinical and educational context [20], but all patients had baseline scores indicating normal-to-mild impairment. The profound decline in group A exceeded typical values reported in postoperative delirium [21] and persisted at 48 hours, suggesting that delirium may exacerbate neuroinflammation post CPB, creating a vicious cycle.

TCD enables noninvasive, continuous hemodynamic monitoring during CPB and is a validated tool for assessing autoregulation and compliance [22]. Measurements by experienced operators exceed 95% reliability [23]; in this study, all data were collected by a single expert. TCD-derived reductions in Vm and elevations in PI correlated strongly with delirium and showed superior predictive sensitivity compared to conventional metrics like MAP. Elevated PI suggests increased distal CVR under stable perfusion [24], and consistent findings across studies support its role in indicating hypoperfusion and delirium risk [25,26]. Of note, flow differences emerged only after aortic clamping, not anesthesia, suggesting minimal influence from our anesthetic protocol, contrary to some reports of propofol-induced CVR elevation [27], possibly due to preserved autoregulation under mild hypothermic CPB.

This study has several limitations. Its single-center design may introduce selection bias, requiring multicenter validation. Although we excluded patients with preoperative cognitive impairment, the lack of biomarker or neuroimaging data prevents definitive causal inference between hemodynamic changes and neuronal injury. The 48-hour follow-up also limits insight into long-term cognitive outcomes, which is important given evidence that CPB-related deficits can persist for years.

## Conclusions

This prospective cohort study establishes intraoperative cerebral hemodynamic disturbances (quantified by TCD) and prolonged cardiopulmonary bypass duration as independent predictors of postoperative delirium in cardiac valve surgery patients. These findings position TCD monitoring as a clinically actionable tool for real-time detection of cerebral hypoperfusion; consequently, intraoperative management should prioritize minimizing bypass time and maintaining cerebral perfusion through TCD-guided optimization. Future multicenter studies must validate these associations and develop targeted reperfusion strategies to promote neurological recovery.
